# Interferon Response and Viral Evasion by Members of the Family Rhabdoviridae

**DOI:** 10.3390/v1030832

**Published:** 2009-11-09

**Authors:** Elizabeth J. Faul, Douglas S. Lyles, Matthias J. Schnell

**Affiliations:** 1 Department of Microbiology and Immunology, Jefferson Medical College, Thomas Jefferson University, Philadelphia, PA 19438, USA; 2 Department of Biochemistry, Wake Forest University School of Medicine, Medical Center Boulevard, Winston-Salem, NC 27157, USA; 3 Jefferson Vaccine Center, Jefferson Medical College, Thomas Jefferson University, Philadelphia, PA 19438, USA

**Keywords:** rhabdovirus, interferon, rabies virus, vesicular stomatitis virus

## Abstract

Like many animal viruses, those of the Rhabdoviridae family, are able to antagonize the type I interferon response and cause disease in mammalian hosts. Though these negative-stranded RNA viruses are very simple and code for as few as five proteins, they have been seen to completely abrogate the type I interferon response early in infection. In this review, we will discuss the viral organization and type I interferon evasion of rhabdoviruses, focusing on vesicular stomatitis virus (VSV) and rabies virus (RABV). Despite their structural similarities, VSV and RABV have completely different mechanisms by which they avert the host immune response. VSV relies on the matrix protein to interfere with host gene transcription and nuclear export of anti-viral mRNAs. Alternatively, RABV uses its phosphoprotein to interfere with IRF-3 phosphorylation and STAT1 signaling. Understanding the virus-cell interactions and viral proteins necessary to evade the immune response is important in developing effective vaccines and therapeutics for this viral family.

## 1. Introduction

The *Rhabdoviridae* family is comprised of more than 175 different currently classified viruses, which are able to infect vertebrates, invertebrates and plants. Common features shared by all rhabdoviruses are an elongated bullet-like shape and an enveloped virion that contains a single-stranded nonsegmented RNA genome. In addition, rhabdoviral genomes encode a basic set of five structural proteins: a nucleoprotein (N), a phosphoprotein (P), a matrix protein (M), a single trans-membrane glycoprotein (G), and an RNA-dependent RNA polymerase (L). Of note, plant as well as some animal rhabdoviruses can encode up to four additional nonstructural proteins [1]. In principle, these nonstructural proteins could be involved in suppression or evasion of host antiviral responses. However, to date the only known mechanisms involve structural proteins that serve dual functions in both virus replication and suppression of host responses.

Although the viruses classified in the *Rhabdoviridae* family have many similarities, there are also important differences in the way different members interact with the host defense mechanisms. This is presumably due to the different defense mechanisms within their hosts. Whereas in plant and insect cells micro RNA (miRNA) probably play a major role in host defense mechanism against rhabdoviral infections [2,3], in higher vertebrates the production of type I interferon (IFN) and initiation of the innate immune response is critical. Most viral pathogens of higher vertebrates are sensitive to IFN-induced antiviral proteins (see other articles in this issue), and thus, have adapted individual mechanisms to avert the innate immune response. This review will focus on type I interferon induction and evasion by two well-studied animal pathogens rabies virus (RABV) and vesicular stomatitis virus (VSV). These viruses are representative of two major Rhabdovirus genera, *Lyssavirus* and *Vesiculovirus*, respectively. These viruses are quite similar in their mechanisms of replication, but differ markedly in their host range, pathogenesis, and mechanisms of immune evasion.

More than twenty years ago type I IFN production in response to RABV infection, especially at the site of infection, was described [4]. It has also been reported that the high levels of IFN in the serum early following infection with RABV contributes to viral resistance [5]. Furthermore, mice injected with anti-mouse IFN-α/β antibody prior to infection with RABV were more sensitive to the virus than mice injected with a control antibody [4]. Likewise, when the IFN-β gene was cloned into the RABV backbone, it was seen that the recombinant virus had greatly reduced pathogenicity and viral replication in mice, however the immunogenicity of RABV was not decreased [6].

Similar observations for the importance of IFN-α/β induction have been made following VSV infection. Type I IFN has been shown to be critical for resistance of mice to infection with VSV. For example, mice deficient in molecules required for IFN-mediated signaling are more sensitive to VSV infection than wildtype mice [7,8]. Interestingly, wild-type VSV infections do not induce type I IFN production in the central nervous system of mice, however type I IFN is rapidly induced in the periphery of these animals [9], which serves to protect most tissues from viral pathogenicity. Taken together, these findings imply that type I IFN plays a major role in the viral lifecycle of RABV and VSV. Furthermore, it indicates that both viruses have developed mechanisms to interfere with the innate host response in order to establish productive infections in their hosts.

## 2. Genomic Organization and Viral Lifecycle of RABV and VSV

In order to better understand the host innate immune response to RABV and VSV infection, we will first briefly describe the genomic organization and the viral lifecycle. As listed above rhabdoviruses encode a “basic set” of five proteins, which are encoded on a genome that has a gene order of 3′ N-P-M-G-L 5′. In addition, all rhabdoviruses contain a noncoding 3′-leader RNA and a non-transcribed trailer sequence at the 5′ end. The rhabdoviral genes are flanked by intergenic sequences, which are in part conserved between all members of the family [10,11]. Following transcription, the viral proteins are translated from monocistronic mRNAs.

The N protein of rhabdoviruses encapsulates the RNA genome and antigenome into a tight, RNase-resistant core [12]. Only the encapsidated RNA, termed the ribonucleoprotein (RNP), is a functional template for transcription and replication. It is also thought that the level of N protein in the cells regulates the switch from transcription to replication by interactions with the leader RNA (leRNA) sequence [13]. It is speculated that if enough N protein is available within the cell, encapsidation of the leRNA sequence results in the synthesis of a full-length antigenome rather the production of individual viral mRNA, therefore switching transcription to replication [13].

The P protein serves as the non-catalytic subunit to the viral transcriptase complex. Once P is phosphorylated it was proposed to form trimers that are able to bind L and N [14]. However, more recently structural analysis revealed that P is dimmer or tetramer [15–17]. Another function of P is to serve as a chaperone for N and prevent it from aggregation [18,19]. Additionally, RABV P is able to antagonize interferon signaling, as discussed in subsequent sections of this review.

The M protein is able to interact both with the nucleocapsid and with the cellular plasma membrane [20]. Thus, the primary function of a rhabdoviral M protein is to bridge the viral membrane containing envelope and the nucleocapsid core during viral assembly and budding. The balance between transcription and replication is thought to be dependent on the M protein in addition to the quantity of N in the infected cell [21]. A high concentration of VSV M inhibits RNA synthesis *in vitro* [22]. It has been proposed that the association of RNP with VSV M results in the condensation of the RNP molecule, which renders it non-functional for transcription and replication. In addition, M has been seen to have secondary functions following VSV infection that might have been evolved later. Namely, VSV M has the ability to inhibit host defense mechanisms like type I interferon signaling, which will be discussed later in this review.

The fourth gene encodes the G protein, which is the only trans-membrane glycoprotein in this family of viruses. The G proteins trimerize in the ER [23] and the association is further stabilized by lateral interactions among trimers on the virion’s surface [24]. At lower pH values the G protein undergoes a conformational change, which exposes hydrophobic residues allowing for fusion with target membranes [25,26]. It is interesting to note that for both VSV G and RABV G, the pH dependent conformational changes required for fusion are reversible [27,28].

The catalytic subunit of the rhabdoviral polymerase complex is the large (L) protein. In addition to transcription, this large protein is capable of capping and methylating the 5′ end of the mRNA ([29] and [30], respectively) and polyadenylating the 3′ end [31].

As with many viruses, the lifecycle of *Rhabdoviridae* can be divided into three distinct phases: uncoating, transcription/replication, and viral assembly. The first phase initiates when a viral particle binds to the surface of a host cell and enters by endocytosis. Attachment to the host cell requires the G protein. However, it is less clear which cellular molecule(s) interacts with G to mediate viral entry. Of note, the nicotinic acetylcholine receptor [32], neuronal cell adhesion molecule [33] and low-affinity nerve-growth factor receptor (P75NTR) [34] has been suggested as receptors for RABV. On the other hand, VSV G protein appears to interact with negatively charged lipids on the cell surface through a combination of electrostatic and hydrophobic interactions [35–37]. Following receptor binding, rhabdoviruses are endocytosed. Once in an endosome, the viral membrane fuses with the endosomal membrane to release the genome into the cytosol. Fusion of the virus with host membranes requires a change to a lower pH within the vesicle. Consequently, membrane fusion and the release of the capsid into the cytosol can be inhibited by both ammonium chloride and chloroquine [38,39].

In the second phase, virion components are produced. Direct translation of the rhabdoviral genome cannot occur because its RNA is negative sense, and furthermore, direct translation is prevented by the encapsidation of the genome by N [40–43]. Thus, after release into the host cell cytosol the viral polymerase complex contained within the capsid is responsible for the RNA synthesis. The most widely accepted model for transcription of rhabdoviruses is the so-called stop/start model, where the polymerase stops at a signal sequence and restarts transcription at the transcription start signal sequence [44]. Reinitiation of transcription by the polymerase complex for the following gene does not always occur; therefore, attenuation of transcription occurs in a 3′ to 5′ direction [45]. Probably caused by the concentration of N protein, the viral polymerase switches to replication and transcribes a full-length anti-genomic RNA, which is also encapsidated into the N protein and serves a template for the replication of new genomic RNAs .

The last phase of the life cycle is the assembly of the viral components, budding and finally release of the virions. For this process, the RNP-complex must be engulfed in the host cell membrane. It is as of yet unknown how the capsid is transported to the cellular membrane. Budding seems to be at least partially dependent on G, as in the absence of RABV-G RABV is released 30-fold less efficiently [46] and similar finding have been made for VSV [47]. More dramatically, in the absence of RABV M viral titers were reduced as much as 500,000 fold, but this did not greatly affect viral protein expression [48]. Likewise, VSV M plays a role in seizing the endosomal-membrane fusion machinery to facilitate viral budding. This occurs via the PPPY motif near the N-terminus of VSV M, which allows for interaction with the cellular enzyme Nedd4 [49].

## 3. Viral Induced Expression of Type I Interferon

The hallmark response to viral infection is the induction of type I interferon (IFN). This class of cytokines is comprised of several IFN-α genes, a single IFN-β gene, and the more recently added, and less well-defined, genes such as IFN-ω, -ɛ, -τ, -δ, and –κ [50]. All nucleated cells have the ability to both produce type I IFN and also to respond to it via a common heterodimeric receptor [51]. Type I IFNs have been seen to induce an anti-viral state in cells, which makes them resistant to subsequent viral infection [52]. In addition, this group of cytokines helps to initiate the adaptive immune response following infection [6,53,54].

A variety of viral moieties can trigger activation of the IFN-α/β signaling cascade by numerous pathogen pattern recognition receptors (PRRs). In each case however, the signaling culminates in the binding of cytoplasmic transcription factors, namely IFN regulatory factor-3 (IRF-3) and nuclear factor kappa B (NF-κB), to the IFN-β promoter. Following signaling initiation, the C-terminus of IRF-3 is phosphorylated and the proteins dimerize. This dimerization reveals a nuclear-localization signal (NLS) on IRF-3, which allows for transport into the nucleus [55]. Alternatively, cytoplasmic NF-κB can translocate to the nucleus following signal induced ubiquitination and proteasomal degradation of inhibitor of NF-κB (IκB) (for review see [56]). Optimal binding and induction of IFN-β requires the additional binding of c-jun/ATF-2 to the IFN-β promoter. When all three transcription factors complexes assemble onto the promoter it is referred to as the enhanceasome. The enhanceasome aids in the recruitment of CREB-binding protein (CBP)/p300, which promotes the assembly of the transcriptional machinery and RNA polymerase II [57]. Each transcription factor can bind to the IFN-β promoter individually, however with limited affinity. It is generally accepted that the binding of IRF-3 is indispensable for induction, while the binding of NF-κB and c-jun/ATF-2 is not required [58]. Similarly, the transcription of most interferon-α genes requires activation of IRF-7, whose expression is induced in response to interferon-β in many cell types [59].

The upstream signaling mechanisms leading to type I interferon production vary markedly among different cell types depending on the expression of various pattern recognition receptors. The receptors that have been demonstrated to be important for the response to rhabdoviruses include Toll-like receptors (TLR), which are expressed either on the cell surface or in the endosomal compartment, and RIG-I-like receptors, which are expressed in the cytoplasm.

### 3.1. Toll-like Receptor Signaling

Signaling through many Toll-like receptors can induce IFN-α/β. One such receptor is TLR-3, which is located in the endosome and binds double stranded RNA molecules [60]. Following ligand binding, TLR-3 dimerizes and is phosphorylated at a tyrosine residue. It then recruits the adaptor molecule TIR-domain containing adaptor inducing IFN-β (TRIF) [61] which leads to the activation of both IRF-3 and NF-κB by divergent signaling cascades [62].

Negative stranded RNA viruses do not produce double stranded RNA as part of their normal replication cycle, since the negative RNA strand is always encapsidated. However, it is likely that abnormal replication products resulting from errors by viral RNA-dependent RNA polymerases give rise to some level of double stranded RNA in virus-infected cells [63]. Using mice deficient in TLR-3, it was determined that this receptor is dispensable in clearing VSV, as mice remained resistant to infection despite the loss of TLR-3 signaling [64]. On the other hand, TLR-3 signaling following a RABV infection may play a role in the induction of IFN-α/β by infected neurons. Following infection of human postmitotic neurons with RABV, Prehaud *et al.* saw an increased production of IFN-β and TLR-3 mRNAs. Treatment of neurons with poly(I:C), a TLR-3 agonist, generated similar cytokine profile to that which was seen following RABV infection [65]. Furthermore, the expression of TLR-3 on cerebellar cortex tissues of individuals that had died of rabies, but not on an individual that died of cardiac arrest, verify the viral induced expression of TLR-3 in human brains *in vivo* [66]. Although this data did not definitively prove a role for TLR-3 in the innate immune response to RABV, it does provide evidence that TLR-3 signaling could be involved. Interestingly however, TLR3-/- mice have a reduced susceptibility to a pathogenic RABV stain, CVS-11 [67]. This increase in survival may be unrelated to the IFN-α/β response and rather due to the requirement of TLR-3 in forming viral Negri Bodies, which are suggested to be required for viral replication [67,68].

Unlike TLR-3, which has relatively wide tissue distribution, another TLR capable of inducing IFN-α/β, TLR-7, has a much more restricted expression profile. TLR-7 is expressed primarily in different subclasses of dendritic cells, such as plasmacytoid dendritic cells (pDCs), a cell type that can produce the majority of the circulating IFN following a viral infection (for review see [69]). The ligands for TLR-7 are immunomodulatory compounds (ie-imiquimod) [70] or single-stranded RNA molecules [71]. Upon ligand binding, TLR-7 recruits the adaptor protein myeloid differentiation factor 88 (MyD88), which then mediates IFN-α/β production. MyD88 can complex with several proteins, namely TRAF-6 and IRAK-1, and initiate the direct binding of IRF-7 [72–74]. Following recruitment into the complex, IRF-7 is phosphorylated by IRAK-1 and translocates to the nucleus where it binds to the promoters for type I IFNs..

Although the role for TLR-7 signaling following a RABV infection has yet to be determined, it appears that TLR-7 dependant signaling is important in the type I IFN response to VSV. Infection of wild type pDCs with VSV induced the production of both IFN-a and IL-12 p40. However, infection of pDCs from TLR7-/- or MyD88-/- mice resulted in no cytokine production [75]. Thus, indicating that single-stranded RNA derived from VSV is able to trigger TLR-7 signaling. The response of dendritic cells to VSV seems to be restricted to TLR7^+^ DC, as no IFN-α/β production is seen following infection of myeloid DCs that do not express TLR-7 [76,77]. Interestingly however, when myeloid DCs are infected with an M-mutant VSV they are able to induce a type I IFN response, albeit independent of MyD88 signaling [77].

VSV replication is required to activate TLR-7 signaling in plasmacytoid DCs [78,79]. However, as discussed above, VSV replication occurs in the cytoplasm, and thus replication intermediates, such as ssRNA, are spacially removed from endosomal TLR-7. It was hypothesized that autophagy may play a critical role in bringing ligand and receptor into the same subcellular compartment following VSV infection. To address this possibility, Lee *et al.* treated pDCs with inhibitors of autophagy, either Wortmannin or 3-MA, prior to infection. It was seen that the inhibitors had no effect on VSV infection rates, but that they did inhibit IFN-α production in a dose-dependent manner [78]. Thus, indicating a critical role for autophagy in initiating TLR-7 signaling in VSV infected cells.

TLR-4 is also able to induce both IRF-3 and NF-κB signaling in response to extracellular signals. Although TLR-4 is generally associated with its ability to bind bacterial products, namely lipopolysaccharide, it has been seen that VSV G can trigger IFN-α/β production via TLR-4 signaling [80]. Furthermore, this type I IFN response may be important to disease outcome because while more than 50% of the TLR-4-/- mice succumbed intravenous VSV challenge, 100% of wild type mice survived [80].

### 3.2. RIG-I-like Receptor Signaling

In addition to TLRs, cells can also induce type I IFN via a cytoplasmic pattern recognition receptor family, the RIG-I-like receptors (RLR). Like TLR signaling, RLR signaling converges on NF-κB and IRF-3 to induce a robust IFN-α/β response. Two of these receptors, namely retinoic acid-inducible gene-I (RIG-I) and melanoma differentiation-associated gene-5 (Mda-5), have been reported to be important in the recognition of RNA viruses [81]. Signaling by RIG-I and Mda-5 is mediated through the mitochondria-bound protein interferon beta promoter stimulator-1 (IPS-1), which is also referred to as MAVS, Cardif, or VISA [82–85]. Of note, IPS-1’s localization in the mitochondria is critical for activation of IRF-3 and NF-κB [84]. IPS-1, with the help of TRAF-3, facilitates the activation of two kinases, TANK-binding kinase-1 (TBK1) and IKKi (also known as IKKe), that go on to activate IRF-3 [82,86].

Whereas Mda-5 signaling has been seen to be important in the IFN-α/β response to picornaviruses [87], RIG-I signaling is critical in generating type I IFN following a rhabdovirus infection. Although the L protein of rhabdoviruses has a capping function [29], the leader RNA remains unmodified [88,89]. Thus, viral transcription generates some single-stranded RNA molecules bearing a 5′ triphosphate, which is one of the known ligands for RIG-I. Following infection with a recombinant RABV that expressed very little P, IFN-β promoter activity was reported, however transfection of a dominant-negative mutant RIG-I molecule prior to infection abrogated the response [90]. Thus indicating, that RIG-I was responsible for the IFN-α/β response following infection. Interestingly, viral replication does not seem to be required for IFN induction as transfection of RNA isolated from RABV infected cells was sufficient to induce a response. On the other hand, the presence of 5′-triphosphate RNA was observed to be essential for eliciting a type I IFN response as dephosphorylated viral RNA induced no IFN-β promoter activity [90].

RIG-I signaling has also been reported to be important for generating IFN-α/β following infection with VSV. Following intranasal infection with VSV, RIG-I expression in microglia and astrocyte cultures is upregulated [91]. It was also seen that unlike in wildtype cells, mRNA for IFN-β and CXCL10 was not upregulated in mouse embryo fibroblasts (MEFs) from RIG-I-/- mice [92]. Additionally following infection with VSV, RIG-I-/- MEFs had a 1.5-log increase in viral titers compared to wildtype cells [92]. This phenotype was reproduced *in vivo*, as RIG-I-/- mice were significantly more susceptible to an intranasal infection with VSV than wildtype littermate controls [87]. Furthermore, it was determined that RIG-I, but not Mda-5, was responsible for inducing the type I IFN response after VSV infection [87].

## 4. Type I Interferon Induced Antiviral State Following Infection

As mentioned above, the receptor for type I IFN is expressed on a wide range of cells. Additionally, IFN-α/β can act in both an autocrine and paracrine manner to induce an anti-viral state in the cell. When IFN-α/β binds to its heterodimeric receptor oligomerization and a subsequent conformational change occurs allowing members of the Janus kinase (JAK) family to phosporylate the IFNAR1 subunit. The phosphorylated receptor subunit can then act as a docking site for members of the signal transducers and activators of transcription (STATs), which are in turn phosphorylated by JAKs [50]. Once STAT-1 and STAT-2 are phosphorylated they dimerize and create a novel NLS, which allows for their transport into the nucleus. The STAT1/2 complex forms a heterotrimer with IRF-9 either in the nucleus or at the receptor, making what is referred to as the ISGF3 complex. The canonical activation of IFN-stimulated genes (ISGs) occurs by ISGF3 binding to the IFN-stimulated response element (ISRE) present in the promoter of most ISGs [50]. Of note, all of the components that make up the ISGF3 are IFN-inducible.

Following treatment with IFN-α/β, the transcript frequency for numerous genes increases [93]. Many of these gene products encode proteins or transcription factors involved in the aforementioned signaling cascades, thus acting as an amplification loop to increase the amount of type I IFN produced. Other genes encode for proteins with direct antiviral functions. Here we will only discuss the antiviral gene products that have been shown to play a role in a RABV or VSV infection, however that does not imply that those genes not discussed do not play a role in the innate response to these or other rhabdoviruses.

One such anti-viral protein is the Mx family of GTPases, which are highly induced in response to type I IFN. The main viral target for these proteins is nucleocapsid-like structures and after binding they appear to sequester the viral proteins and interfere with proper localization [94]. It was seen that expression of bovine Mx1 can reduce viral titers and decrease N protein levels in rabies infected VERO cells [95]. Likewise, VSV replication is inhibited by a wide variety of Mx proteins *in vitro* [96–98]. The human MxA protein was seen to inhibit both viral mRNA and protein levels following infection with VSV for 5 hours [98]. Furthermore, transgenic mice that express high levels of bovine Mx1 are resistant to a lethal challenge of VSV [99].

Another IFN-induced gene that affects both RABV and VSV is promyelocytic leukaemia (PML). PML is expressed in the nucleoplasm and nuclear bodies (NB) of the cell. Following IFN-α/β treatment, enhanced PML protein expression is seen and the NB size markedly increases [100,101]. The anti-viral properties of PML are still unclear, however it is known that expression is essential for type I IFN induced apoptosis [102] and that PML has growth suppressing properties [100]. Like Mx proteins, it was observed that overexpression of PMLIII induced resistance to VSV infection, by inhibiting mRNA and protein synthesis [103]. Of note, complete inhibition by PML was only seen following infections at a low multiplicity of infection [103]. Furthermore, following type I IFN treatment MEFs derived from PML-/- mice had similar resistance to VSV infection as seen in wildtype cells [101]. The anti-viral role of PML following a RABV infection has not, as of yet, been experimentally proven. Rather, an anti-viral role is hypothesized due to RABV P protein’s ability to disrupt NB organization and increased rabies virus replication, as seen by higher protein level expression and 20 times more virus, in PML-/- MEFs [104]. The details of RABV induced PML inhibition will be discussed in detail in the next section.

Although the antiviral effects induced by Mx/PML is similar for RABV and VSV, there are a number of antiviral proteins that have only been reported to play a role following VSV infection, namely PKR and ISG15. PKR and ISG15 have completely unique mechanisms to alter the host response to a viral infection. PKR is constitutively expressed in all tissues, however it is activated and upregulated by either dsRNA or type I IFN treatment. PKR phosphorylates eukaryotic translational initiation factor (eIF)-2α, which results in the sequestering of eIF2-B. Thus, translation initiation is halted [105]. Mice deficient in PKR are much more sensitive to intranasal VSV infections than wildtype mice [106–108].

Alternatively, ISG15 is an ubiquitin homologue that activates its substrates, rather than targeting them for degradation [109]. ISG15 substrates include, among others, signaling components in the JAK/STAT and RIG-I pathways, MxA, and PKR [110]. The requirement for ISG15 in the innate response to VSV is still unknown, due to conflicting results. Lu *et al.* noted that ISG15-/- MEFs are more permissive to VSV replication, however there was no notable differences in the knockout cells’ sensitivity to the antiviral effect of IFN-α/β [109]. On the other hand, using a knockout mouse that was only deficient in ISG15 conjugation, not free ISG15, there was no difference in the susceptibility of cells to VSV in an IFN-protection assay [111]. Of note, ISG15 is regulated by USP18, which catalyzes its hydrolysis to remove it from conjugated proteins [112,113]. Interestingly, USP18-/- mice show an increased survival following intracerebral infection with VSV as compared to wildtype mice [114].

## 5. Rhabdoviruses IFN-α/β Antagonism Mechanisms

In order for an effective infection to occur, RABV and VSV must antagonize the interferon signaling cascade and block induction of antiviral molecules. However, RABV and VSV have completely different mechanisms by which they antagonize the immune response. RABV specifically targets signaling molecules in order to block IFN induction and subsequent responses. On the other hand, VSV takes a more global approach and inhibits host-cell transcription and translation. In both cases the IFN antagonism is due to viral proteins that play key roles in virus replication and have separate functions involved in inhibition of host antiviral responses, the RABV P protein and the VSV M protein.

### 5.1. Rabies Virus IFN Escape Mechanisms

A key viral goal is to control, and minimize, the innate immune response following infection. As type I IFN production is critical in an innate response, one mechanism by which a virus can curtail the immune response is by diminishing IFN-α/β production. RABV intervenes with the multiple IFN induction pathways where most of them converge, namely the activation of IRF-3. The P protein of RABV prevents IRF-3 phosphorylation by the TBK1, thus suppressing IFN-α/β production [115]. It seems as though following infection a race between host and virus begins, and thus the expression level of RABV P is critical for antagonizing IFN-α/β responses. Brzozka *et al.* saw that following infection with a recombinant RABV that expressed only low levels of P the virus was no longer able to suppress IFN-β production [115].

In addition to antagonizing IFN induction, RABV also has a mechanism to inhibit type I IFN signaling. Thus, virus infected cells are resistant to the actions of IFN, regardless from where the cytokine originated. Vidy *et al.* reported that following a RABV infection STAT-1 does not accumulate in the nucleus. However, RABV P neither induces STAT-1 degradation nor does it interfere with STAT-1 phosphorylation; thus, it was concluded that IFN-induced transcriptional responses are prevented [116]. Recently, Moseley *et al.* suggested another mechanism by which RABV P can interfere with STAT-1 signaling. It was shown that RABV P can facilitate a shift from conventional interaction of STAT-1 and microtubules to a microtubule-inhibited interaction, thus preventing STAT-1 nuclear import [117]. Furthermore, it is known that only tyrosine-phosphorylated STAT-1 and -2 are bound by RABV P [118]. This data indicates that IFN-induced JAK/STAT activation is required for P binding to STAT-1 or -2. It was also determined that binding of RABV P to STAT-1 and -2 requires the 10 C-terminal amino acids in RABV P [118].

Of note, RABV P is expressed not only as full-length P protein but also in four N-terminal truncated forms (P2-P5) that are synthesized from internal start codons [119]. While all five P proteins contain a NLS, only two forms, P and P2, have the nuclear export signal (NES) found in the N-terminus [119]. Therefore, the P3, P4 and P5 forms are found primarily in the nucleus. Interestingly, P not only retains STAT-1 in the cytoplasm but also has the ability to bind the transcription factor in the nucleus via P3–P5 [120]. Thus, preventing ISGF3 complexes from binding the ISRE promoter in the nucleus [120].

Lastly, there is evidence that RABV also interferes directly with IFN-induced antiviral molecules, namely PML. The role that PML potentially plays in orchestrating an immune response to RABV or impacting viral replication is not yet clear. However, it has been shown in RABV infected cells that RABV P retains the PML protein in the cytoplasm and that the P3 isoform disrupts nuclear bodies organized in the nucleus [104]. Thus, disrupting PML’s association with nuclear bodies and potentially critical functions in nuclear trafficking, including viral defense and apoptosis [121]. Again, the mechanism by which PML nuclear bodies function in viral infection is still not understood, but the binding and retention of PML by RABV P provides some indication that PML may have an antiviral function [104,122].

### 5.2. Vesicular Stomatitis Virus IFN Escape Mechanisms

The matrix protein of VSV is crucial in averting the host IFN-α/β response. VSV M works to interrupt cellular transcription pathways and also to block mRNA export from the nucleus, both of which function to antagonize the host immune response. The ability of M to inhibit host gene expression is genetically separable from its role in virus structure and assembly. Several M protein mutant viruses have been identified that are defective in the inhibition of host gene expression, but otherwise replicate and assemble as effectively as wildtype VSV [123–126]. Like RABV P, VSV M mRNA is also translated into N-terminally truncated proteins from alternative initiation codons [127]. These truncated proteins also function to inhibit host gene expression, although they are not capable of functioning in virus assembly.

VSV M alone is sufficient to shut down cellular transcription. Expression of VSV M in transfected cells inhibits both the host genes and the transfected plasmid transcribed by polymerase I, II, or III [128,129]. M is also capable of inhibiting nuclear-cytoplasmic RNA transport in the absence of other VSV components [130,131]. The general inhibition of host cell transcription leads to significant cytotoxicity in VSV infected cells. Of note, transfection of VSV M is sufficient to induce cytopathic effects in cells due to the induction of apoptosis [132,133].

Like RABV P, VSV M does not have enzymatic activity, but instead it probably inhibits host antiviral responses by binding to host factors and interfering with their normal function. Thus far, the only host proteins whose binding to VSV M is correlated with the inhibition of host gene expression are Rae1 and the nucleoporin Nup98 [131,134]. These two proteins are present as a complex in cells that is thought to be involved in mRNA transport [135,136], and the binding of M to Nup98 has been shown to be indirectly mediated through Rae1 [134]. While binding of M protein to the Rae1-Nup98 complex is likely to be responsible for the inhibition of nuclear-cytoplasmic RNA transport, this is not due simply to the inhibition of Rae1 function, since Rae1 is not essential for mRNA transport. For example, silencing Rae1 expression in mammalian cells by siRNA [137,138] or deletion of Rae1 in mouse embryo cells [139] does not inhibit cellular gene expression. A further question is whether the binding of M to Rae1 is also responsible for the inhibition of host transcription, as well as nuclearcytoplasmic transport, or alternatively, whether there are additional host factors that are targets for VSV M.

## 6. Conclusions and Implications for Disease Treatment

Here we have discussed the different ways by which type I IFN is induced by RABV and VSV, the host antiviral proteins that inhibit the viral lifecycle, and the mechanisms by which RABV and VSV antagonize IFN-α/β pathways. While both rhabdoviruses can induce IFN-α/β by the RIG-I pathway, it seems they use different TLRs. VSV can signal through TLR-7 and TLR-4, while TLR-3 is dispensable for inducing IFN. On the other hand, signaling via TLRs is less well studied following a RABV infection but there is data to indicate that TLR-3 may play a role. Both RABV and VSV are sensitive to Mx GTPases and PML, while only VSV seems to be affected by PKR and ISG15. Lastly, these two rhabdoviruses have strikingly different mechanisms to antagonize IFN signaling. RABV specifically targets IRF-3 to minimize IFN-α/β production and also inhibits the antiviral response in infected cells by binding STAT-1 and -2 and disrupting PML nuclear bodies. On the other hand, VSV inhibits cellular transcription pathways and blocks mRNA export to prevent an antiviral response.

Understanding the host response to VSV and RABV is important for developing vaccines against rhabdoviral induced disease but also for developing potential rhabdoviral vaccine vectors. A vaccine strain of RABV expressing IFN-β greatly reduces viral pathogenesis and replication but not the immunogenicity of the vector [6]. Thus, providing a potentially self-limiting and highly immunogenic vaccine vector. Additionally, both RABV and VSV based vaccine vectors have been shown to be efficient vaccines against pathogenic SHIV_89.6P_ challenge [140,141]. Furthermore, VSV has recently been considered for potential use as an oncolytic therapy agent. Many transformed cells have impaired IFN pathways [142], thus VSV can replicate to a much greater extent in those cells than normal cells that have intact IFN signaling. In fact, it has been reported that transformed cells are more susceptible to VSV infection then normal cells following IFN-α/β treatment [143,144]. Thus, ongoing efforts to understand the type I IFN response to rhabdoviruses have obvious implications for improving current vaccines and therapies.
